# Advanced Bending and Forming Technologies for Bimetallic Composite Pipes

**DOI:** 10.3390/ma18010111

**Published:** 2024-12-30

**Authors:** Hui Li, Yingxia Zhu, Wei Chen, Chen Yuan, Lei Wang

**Affiliations:** School of Mechanical Engineering, Jiangsu University, Zhenjiang 212013, China; 2212203054@stmail.ujs.edu.cn (H.L.); chen_wei@ujs.edu.cn (W.C.); 2212303071@stmail.ujs.edu.cn (C.Y.); 2212303045@stmail.ujs.edu.cn (L.W.)

**Keywords:** bimetallic composite pipe, bending and forming, forming defects, state of the art

## Abstract

Bimetallic composite pipes, as critical components, effectively integrate the superior properties of diverse materials to meet the growing demand for lightweight, high-strength, and corrosion-resistant solutions. These pipes find extensive applications in petrochemical, power generation, marine engineering, refrigeration equipment, and automotive manufacturing industries. This paper comprehensively reviews advanced bending and forming technologies, with a focus on challenges such as wrinkling, excessive wall thinning, springback, cross-sectional distortion, and interlayer separation. The review combines theoretical analysis, experimental findings, and numerical simulations to provide insights into defect prevention strategies and process optimization. It also evaluates emerging technologies such as artificial neural networks and intelligent control systems, which demonstrate significant potential in enhancing bending accuracy, reducing defects, and improving manufacturing efficiency. Additionally, this work outlines future research directions, emphasizing innovations required to meet the stringent performance standards of bimetallic composite pipe components in high-end applications.

## 1. Introduction

Bimetallic composite tubes, as lightweight structural components, effectively address specific challenges faced by conventional pipes. Unlike traditional pipes, which often suffer from material waste and rework due to surface and subsurface flaws (e.g., cracks, trapped air bubbles, and pits) [1,2], bimetallic composite tubes offer enhanced manufacturing precision through advanced bonding and forming techniques. Their dual-material construction minimizes issues like non-uniform thickness and circularity by optimizing the compatibility and properties of inner and outer layers. Moreover, these tubes reduce eccentricity by leveraging tightly controlled forming processes, ensuring structural integrity and reliability in demanding applications such as the petrochemical industry [3], energy power generation [4], ocean engineering [5], refrigeration equipment [6], and the automotive industry [7]. Similar advancements have been made in other bimetallic components, such as bimetallic bolts, which utilize dual-material properties to improve mechanical performance and resistance to harsh environments [8]. While their production demands meticulous control, the ability to integrate multiple material properties in a single component represents a significant improvement over conventional single-material solutions, providing superior strength, corrosion resistance, and adaptability to complex geometries.

The manufacturing processes for bimetallic composite pipes can be broadly categorized into two main types: mechanical bonding and metallurgical bonding. Mechanical bonding processes include drawing, rolling, extrusion, expansion, and spinning, which rely on external forces to join the inner and outer tubes, forming a compact structure. Metallurgical bonding, on the other hand, utilizes the physical and chemical interactions of materials at high temperatures, with key techniques including centrifugal casting, solid-liquid casting, and explosive welding. Mechanical bonding is characterized by high forming efficiency and adaptability, while metallurgical bonding achieves superior interfacial bonding strength, making it suitable for producing composite pipes with high-performance requirements.

To accommodate varying specifications, shapes, materials, and molding tolerances, a range of bending methods for bimetallic composite tubes has been developed. These methods can be categorized based on several criteria. First, considering the forming temperature, the bending process is classified into cold bending and hot bending [9,10]. Second, based on loading conditions, the methods include pure bending, compression bending, push-pull bending, CNC bending, laser bending, and free bending [11,12,13,14,15,16]. Third, from a material perspective, bimetallic composite tubes encompass combinations such as copper/titanium, aluminum/steel, and aluminum/magnesium [17,18,19]. Fourth, regarding the shape of the tube, these methods apply to tubes with round, rectangular, and other shaped cross-sections [20,21]. Finally, in terms of bending difficulty, tubes with a large relative radius are easier to bend, whereas those with a small relative bending radius present more challenges [22,23].

During the bending process, the inner and outer layers of composite pipes will be subjected to complex tensile and compressive stress distributions, which may lead to a series of defects or instability phenomena such as wrinkling, excessive wall thinning, cross-section distortion, rebound, and interlayer separation. How to accurately predict and effectively control these phenomena is a long-standing challenge in the field of forming and manufacturing and a focus of researchers’ attention [24,25,26].

At present, for the multiple defects, forming parameters, and process design in the bending and forming of bimetallic composite tubes, researchers have conducted in-depth studies using a variety of methods, which have greatly promoted the technological progress in this field. With the improvement of forming quality requirements for lightweight, high-strength, and corrosion-resistant composite pipe fittings in high-end industries such as petrochemical and offshore engineering, the design of bimetallic composite pipes, control of multiple instabilities, and enhancement of process limits are facing new challenges. To cope with these challenges, this work reviews the common basic problems in bending and forming of bimetallic composite tubes, such as wrinkling instability, wall thickness thinning, interlayer separation, cross-section deformation and rebound, etc. It also evaluates the advantages and disadvantages of the current bending and forming technologies and discusses the future development direction and problems to be solved.

## 2. Bimetallic Composite Pipe Bending Theory Research Progress

The bimetallic composite pipe features a base-liner structure, which consists of two main components: the inner tube and the outer tube. To meet the diverse property requirements for specific application environments, different materials are typically selected for the inner and outer layers. Common materials include aluminum alloys, magnesium alloys, alloy steels, cast iron, and copper alloys, as detailed in Table 1.

Investigations [24,25,26] have shown that the bimetallic composite tube bending process can lead to forming defects such as wrinkling, excessive wall thickness thinning, cross-section distortion, and springback. However, the presence of a double-layer structure results in distinct characteristics that differ from those of single-tube configurations. Consequently, numerous studies have been conducted to examine the bending formation of bimetallic composite tubes, focusing on aspects such as wrinkling, excessive wall thinning, cross-sectional distortion, and springback [9,10,11,12,13,14,15,16,17,18,19,20,21,22,23]. Understanding the occurrence mechanism, accurately predicting, and effectively controlling these defects are the core issues of the bending theory research of bimetallic composite tubes.

### 2.1. Wrinkling

#### 2.1.1. Wrinkling Prediction Study

In the early stage of the study of buckling theory, Hill [33] introduced a sufficient condition for the uniqueness of elastic-plastic materials, which served as a foundational tool for analyzing wrinkling behavior and laid the groundwork for preliminary bifurcation theory. Building on this, Hutchison [34] extended Hill’s theory by applying it to the wrinkling analysis of thin shells, thereby providing critical theoretical support for understanding the buckling behavior of thin shell structures. In the context of the bimetallic composite tube bending process, the interaction and wrinkling behavior of the inner and outer walls can be effectively described using plastic bifurcation theory. This theory, which incorporates the plastic deformation properties of materials, offers a more accurate representation of the buckling and wrinkling phenomena occurring in the inner liner pipe under high bending strains [35]. Yuan et al. [36] further advanced this approach by employing the J2 plastic deformation theory (Equation (1)) to model the inelastic behavior of materials and predict wrinkling in bimetallic composite pipes. Their findings revealed that the bending flexural strain in the inner pipe was significantly lower than that observed in single-layer pipes.

(1) $$\sigma_{ij}=\frac{E_{s}}{\left(1+v_{s}\right)}\left\{\frac{v_{s}}{\left(1-2v_{s}\right)}\delta_{ij}\delta_{kl}+\frac{1}{2}\left[\delta_{ik}\delta_{jl}+\delta_{il}\delta_{jk}\right]\right\}\varepsilon_{ij}$$

where $\sigma_{ij}$ is the stress tensor component, *E_s_* is the modulus of elasticity of the material, *v_s_* is the Poisson’s ratio, and $\varepsilon_{kl}$ is the strain tensor component. $\delta_{ij}$ is the identity matrix filter symbol used in tensor operations, which equals 1 when the subscripts are identical and 0 otherwise. Different combinations of subscripts (e.g., $\delta_{kl}$$\delta_{ik}$$\delta_{jl}$$\delta_{il}$$\delta_{jk}$) are used to represent various stress components in volumetric deformation and shear deformation, respectively.

Unlike bifurcation theory, the energy principle (Equation (2)) has been widely applied due to its simplicity and computational efficiency. For forming processes with relatively simple boundary conditions, instability prediction models can be quickly derived based on energy criteria [37,38]. For example, Zhang et al. [10] investigated the deformation behavior of ultra-thin-walled tubes under the hydraulic push-pull bending process. Through finite element simulations and experimental validation, they demonstrated the effectiveness of the energy principle in wrinkling prediction. Specifically, by analyzing the variation in the system’s total energy during deformation, the critical conditions for wrinkling were determined. When the internal pressure was insufficient, the inner wall of the tube exhibited significant fluctuations in wall thickness, resulting in pronounced wrinkling. By applying appropriate internal pressure, wrinkling could be effectively suppressed, leading to a more uniform wall thickness distribution during the forming process.

(2) $$U_{\min}=W_{ex}$$

where *U_min_* is the minimum energy required for wrinkling, *W_ex_* is the plastic deformation energy in the deformation zone, and wrinkling occurs when Wex reaches a critical point.

Naderi [39] developed an energy-based 3D finite element model for the prediction of bending wrinkling of thin-walled tubes based on the principle of energy, considering geometrical nonlinearities, material defects, inelastic material behavior, and interfacial contact, which was based on a carbon steel/stainless steel composite tube. It was found that geometrical and thickness defects lead to an increase in the probability of the occurrence of wrinkles in the inner tube.

#### 2.1.2. Influencing Factors and Control of Wrinkling

In the process of bending composite pipes, effective strategies for controlling wrinkles encompass the selection of appropriate materials, optimization of the bending process, implementation of mandrel filling techniques, and enhancement of the accuracy in initial preparation (Table 2).

Currently, although the wrinkling phenomenon that occurs during bending of bimetallic composite tubes is well understood, its effective avoidance remains a major challenge to improve the bending limit and to achieve accurate forming, especially in composite tube applications with small relative bending radius. The literature [22] points out that the increase in the diameter difference between the inner and outer materials of bimetallic composite tubes, as well as the change in the contact stress distribution between the inner and outer layers, and the expansion of the critical wrinkling region, increase the risk of wrinkling significantly. During the bending process of composite pipes, the inner and outer materials exhibit distinct stress and strain responses, which can lead to challenges in maintaining structural integrity. A critical issue arises from the delamination or separation at the contact interface between the inner and outer materials, which significantly contributes to the formation of wrinkles. Therefore, enhancing the control and stability of the contact interface between these materials is essential to mitigate the risk of wrinkling and ensure the mechanical performance of composite pipes.

### 2.2. Excessive Wall Thickness Reduction and Cross-Section Distortion

**Figure 1:**
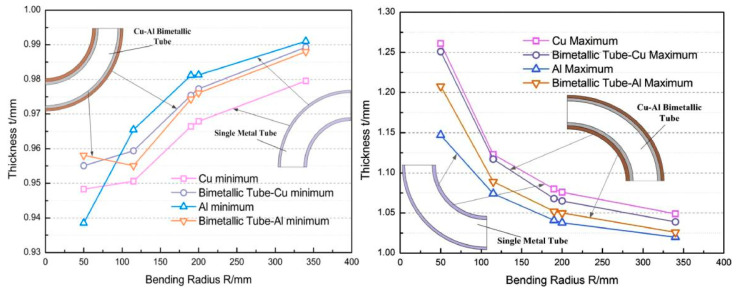
Wall thickness variation of the tube under different bending radii [47].

During the bending process of composite pipes, excessive wall thinning often occurs, primarily due to the uneven distribution of stress between the inner and outer wall materials. As shown in Figure 1, the inner wall is subjected to compressive stress, which can result in localized yielding and plastic deformation of the material. Simultaneously, the outer wall experiences tensile stress, leading to localized deformation and stretching. This disparity in stress distribution between the inner and outer walls is the fundamental cause of the wall thinning phenomenon observed during bending molding. This non-uniformity in stress distribution not only leads to wall thickness thinning but may also cause distortion of the cross-section. The prediction of wall thickness thinning and cross-section distortion is influenced by material properties, geometry, and process parameters, so researchers have continued to explore the laws that influence them to achieve more precise control. These research results provide an important theoretical basis for improving the molding process of composite pipes, enhancing product quality, and ensuring structural reliability.

#### 2.2.1. Theoretical Research on Wall Thickness Thinning and Cross-Section Distortion

**Figure 2:**
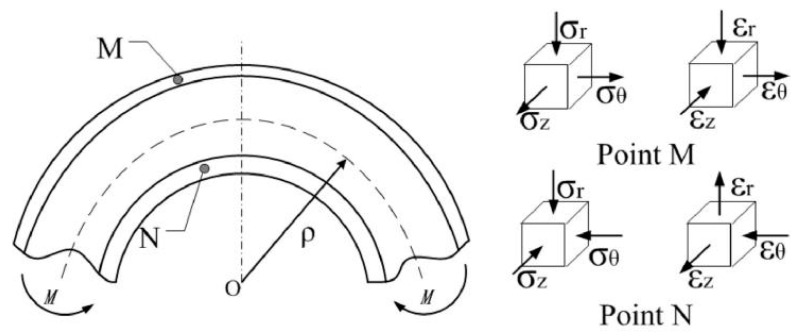
Pipe bending process: external wall structure diagram [47].

Tang et al. [48] pointed out that during the bending process, the outer wall of the tube undergoes tensile thinning, while the inner wall experiences compressive thickening (as shown in Figure 2). To accurately predict the variation in stress distribution during bending, mesh convergence analysis plays a critical role in finite element simulations. By progressively reducing element size, mesh convergence analysis evaluates the relationship between estimation errors and mesh size, thereby yielding precise stress distribution results [49]. Studies have shown that when the element size reaches a certain threshold, the error in the calculated stress distribution significantly decreases, and prediction accuracy improves markedly. The mesh convergence curve effectively validates the reliability of the model and the accuracy of the computational results. Additionally, the necking instability of the material is closely related to wall thickness variation during the bending process. During tension, localized stress concentration leads to necking, which manifests as localized thickening or thinning during bending. By comparing experimental calibration with model predictions, the relationship between stress distribution and wall thickness variation can be further validated, and model parameters can be optimized to enhance prediction accuracy. Moreover, Guo et al. [47] proposed a wall thickness variation prediction model based on plasticity theory (Equation (3)). Incorporating Hencky’s stress–strain relationship, the model reveals the coupled effect of material properties on wall thickness variation and stress distribution. This model enables prediction of the overall wall thickness variation of composite pipes and elucidates the coupling relationship between the thickness changes of the inner and outer layers, as well as the shift in the strain-neutral layer during free bending.

(3) $$0=\frac{4}{3}\int_{\rho_{a}}^{\rho_{N}}\mathrm{sgn}\left(\delta_{1}\right)\cdot Y_{1}\left[{\left(\frac{\Delta t_{1}}{t_{10}}\right)}^{+}\right]d\rho /\rho +\frac{4}{3}\int_{\rho_{N}}^{\rho_{b}}\mathrm{sgn}\left(\delta_{2}\right)\cdot Y_{2}\left[{\left(\frac{\Delta t_{2}}{t_{20}}\right)}^{+}\right]d\rho /\rho$$

where ρ is the radial position, $\rho_{a}$ is the inner radius, $\rho_{b}$ is the outer radius, $\rho_{N}$ is the radius of the neutral layer, $\mathrm{sgn}\left(\delta_{i}\right)$ is the sign function, *Y*_1_ and *Y*_2_ are the flow stress functions of copper and aluminum, respectively, $\Delta t_{1}$ and $\Delta t_{2}$ are the thickness variations of copper and aluminum, respectively, and *t*_10_ and *t*_20_ are the initial thicknesses of copper and aluminum, respectively.

When analyzing the cross-sectional flattening of single-layer tubes, traditional methods usually treat them as elliptical [50], focusing only on the outermost layer at the bending site and ignoring the cross-sectional shape between the outermost layer and the geometric center. However, in the case of bimetallic tubes, the deformation trend of each layer is often inconsistent, and the coupling between the layers leads to a deformation behavior that cannot follow the original mechanical properties. Fu [51] innovatively proposed a physics-driven B-spline curve fitting method to characterize the full cross-sectional deformation of Cu-Al bimetallic tubes during the rotary draw bending process. In this process, the characteristic curve of cross-sectional deformation can be described using the B-spline fitting method. The equation for the characteristic curve $h\left(u\right)$ on the outer side of the cross-section is as follows:

(4) $$h\left(u\right)=\sum_{j=0}^{4}d_{j}N_{j,3}\left(u\right),u\in \left[0,1\right]$$

Here, $d_{j}$ represents the control points, $N_{j,3}\left(u\right)$ is the third-order B-spline basis function, and the distribution of the control points is closely related to the variation in the outer wall thickness. The equation for the characteristic curve on the inner side of the cross-section is expressed as follows:

(5) $$h_{i}\left(\theta \right)=r_{0}+t_{0}+\Delta t\cos\left(\theta \right)+f\left(\alpha ,\beta \right)$$

Here, Δt represents the wall thickness variation, θ denotes the positional angle, and $f\left(\alpha ,\beta \right)$ is the fitting function coupled with curvature and contact pressure.

**Figure 3:**
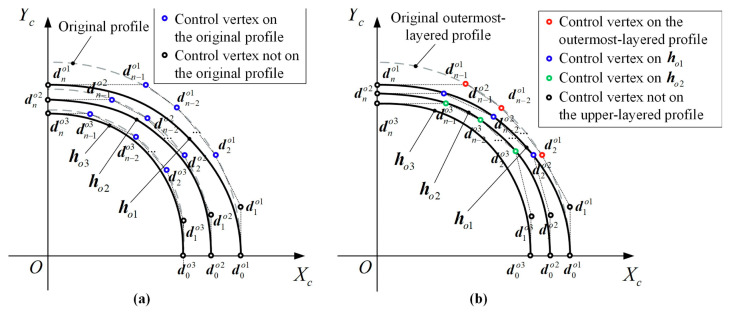
Setting of control vertices according to the convex hull property [51]. (**a**) Control vertices on original profile; (**b**) Control vertices on upper-layered profile.

Figure 3 clearly illustrates the fitting of the outer and inner characteristic curves, particularly highlighting the distribution characteristics of wall thickness variation. The curves accurately describe the geometric features of cross-sectional deformation during the bending process by capturing the coupling relationship between control points and positional angles. Through the application of physical constraint conditions and the principle of energy minimization, the B-spline fitting method achieves precise modeling of the full cross-sectional deformation.

#### 2.2.2. Influence and Control of Wall Thickness Reduction and Cross-Section Distortion

In the composite pipe bending process, effective control of wall thickness reduction and cross-section distortion strategies include the selection of material parameters, the use of mandrel filling, process parameters, and bending method optimization. The relevant studies are shown in Table 3.

These research results provide a solid theoretical basis for improving wall thickness thinning and cross-section distortion of bimetallic composite tubes in the bending process. Although neither can be completely avoided in the bending process, these instabilities can be effectively controlled by the above optimized conditions, keeping them within acceptable tolerances. This ensures the quality and reliability of the composite tube.

### 2.3. Springback

In the bending process of bimetallic composite pipes, springback refers to the shape deviation caused by the release of residual stress after the removal of external forces. Springback theories are typically based on the following assumptions: the material is considered an ideal elastoplastic body, the elastic stage follows Hooke’s law, and the plastic stage conforms to specific hardening laws. However, in practical applications, the strain-hardening effect of materials significantly influences springback behavior. Moreover, traditional theories assume a fixed neutral layer position. However, for bimetallic composite pipes, the neutral layer undergoes significant displacement due to the differing elastic moduli and yield strengths of the inner and outer materials, leading to reduced prediction accuracy. Additionally, conventional springback theories often neglect the effects of strain rate and temperature. In actual production, particularly during high-speed bending or when dealing with temperature-sensitive materials (such as aluminum and magnesium alloys), variations in strain rate and temperature significantly impact springback behavior. These deviations in assumptions limit the applicability of theoretical models under complex conditions. Therefore, in engineering applications, experimental calibration and finite element simulation are essential for more accurate springback prediction and control, ensuring that the forming quality meets practical requirements.

#### 2.3.1. Research on Springback Theory

**Figure 4:**
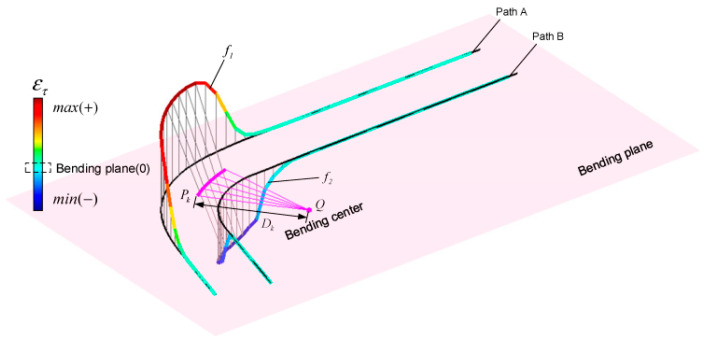
Process diagram of neutral layer shifting extraction [14].

In the study of single pipe springback theory, Al-Qureshi [61] derived the elastic-plastic deformation equation during bending, highlighting that the location of the elastic-plastic transition within the cross-section significantly influences the required bending force, springback degree, and residual stress after unloading. This theory assumes a material model without strain hardening, simplifying the analysis but limiting its ability to reflect real-world strain-hardening behavior during bending. To address this, some studies [62] incorporated a hardening law in the form of a power function into the analytical model, enabling more accurate predictions of springback by accounting for material strain hardening. For composite pipes, traditional single-pipe springback theories are insufficient due to differing material properties across layers. Zhang [11] introduced the concept of a composite elastic modulus *Ec* and neutral layer shift *Sε*, as shown in Equation (6), to enhance the springback model. This approach assumes that the neutral layer shift is a critical factor and simplifies its behavior for calculation, achieving better agreement between theoretical predictions and experimental results (Figure 4). However, such assumptions, including linearized hardening and uniform material properties, limit the applicability of these models under non-ideal boundary conditions or non-uniform material distributions commonly encountered in industrial applications, emphasizing the need for further refinements through experimental calibration and advanced simulations.

(6) $$D_{\varepsilon }=\frac{D_{k}+D_{k+1}+\cdots +D_{k+m}}{m+1},k\in N^{*},k\leq n,m\in N^{*}$$

(7) $$S_{\varepsilon }=R_{0}-D_{\varepsilon }$$

where $D_{\varepsilon }$ is the neutral layer shift, $D_{k}$ is the distance from the neutral layer position to the bending center, $S_{\varepsilon }$ is the relative displacement of the neutral layer, and $R_{0}$ is the initial bending radius.

#### 2.3.2. Influence and Control of Rebound

Table 4 delineates various significant factors that impact pipe springback, thereby affecting the behavior and specific patterns of springback in pipes. A comprehensive understanding of these factors enables more precise predictions and regulation of springback during the bending process, ultimately facilitating the optimization of processing parameters.

Investigations in the literature [70,71] have investigated the impact of employing a composite tube filled with a plastic mandrel on the resilience angle during rotational bending. The findings indicate that the presence of a plastic mandrel within the composite tube establishes an approximately linear correlation between the resilience angle and the bending angle. Furthermore, the application of the overbending compensation method has been demonstrated to effectively regulate the bending angle, thereby enhancing both the precision and efficiency of the bending process.

The above research results have a key role in realizing the precision bending of composite pipes. However, the current effective control of springback mainly relies on empirical and experimental methods. There are many factors affecting the rebound and the fluctuation in the rebound amount is large, which together lead to the challenging task of accurately predicting the rebound angle.

### 2.4. Interface Strengthening

Interfacial bond strength determines the service performance of bimetallic composite pipes. Therefore, interlayer separation is an important molding defect in bimetallic composite pipes. Defects such as wrinkling of the inner tube, thinning of the outer tube, and cross-section distortion will aggravate the interlayer separation defects to a certain extent. At present, overcoming the interlayer separation defect still relies on improving the interfacial bonding strength during the initial preparation of bimetallic composite pipes. The initial preparation methods of bimetallic composite pipes mainly include two types: mechanical bonding and metallurgical bonding.

#### 2.4.1. Control of Interlayer Separation in the Mechanical Composite Process

**Figure 5:**
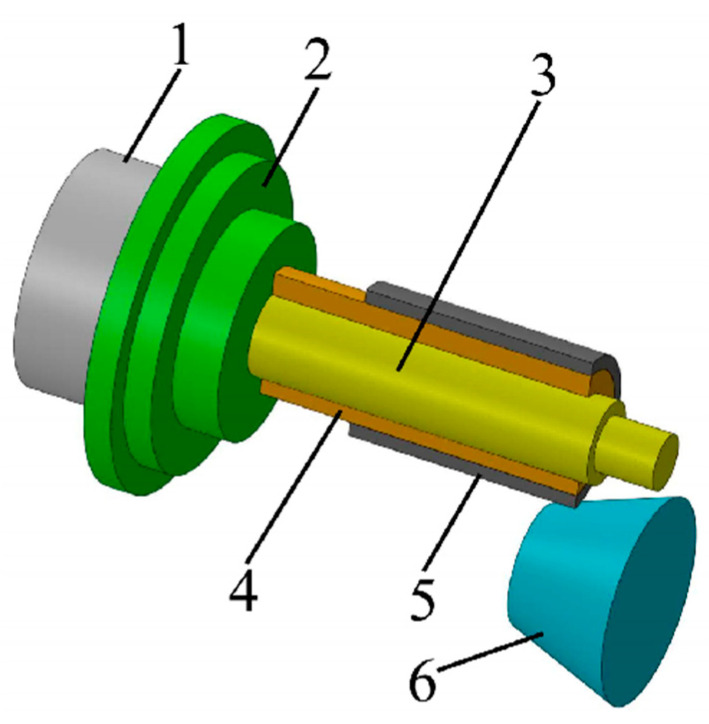
Spinning process (1, spindle; 2, flange; 3, mandrel; 4, inner tube; 5, outer tube; 6, spinning wheel).

**Figure 6:**
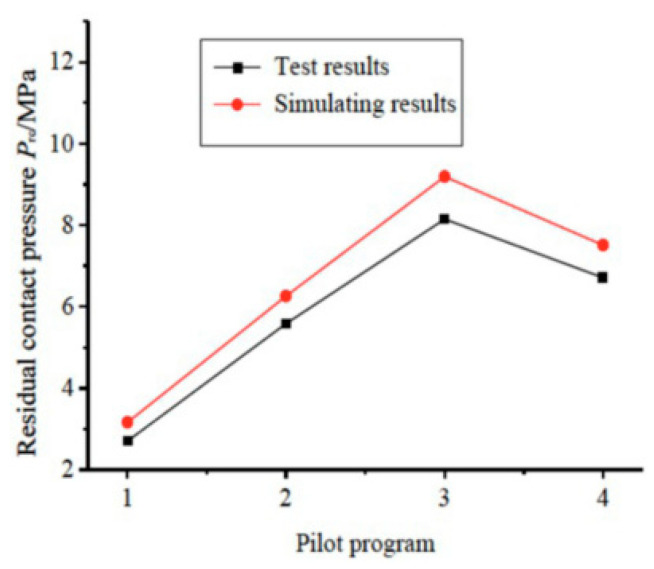
Comparison of residual contact pressure [29].

By optimizing spinning parameters [25,39,72], such as the press-in volume, feed rate, and roller angle, the interfacial bonding strength in spinning processes can be significantly enhanced. Residual contact pressure serves as a critical factor in preventing interlayer separation (Figure 5). Maximizing the residual contact pressure during spinning enables synergistic deformation of the inner and outer materials during bending, mitigating stress concentration and slip at the interface. Furthermore, studies on 20/316L bimetallic composite pipes have demonstrated that when the indentation depth is 0.14 mm, the orientation angle is 2.5°, and the feed rate is 0.3 mm/rev, residual contact stress is effectively improved, ensuring interfacial bonding quality. As illustrated in Figure 6, these optimized parameters facilitate a uniform and higher residual contact stress distribution at the interface, significantly enhancing the stability and performance of composite pipes during subsequent bending processes.

**Figure 7:**
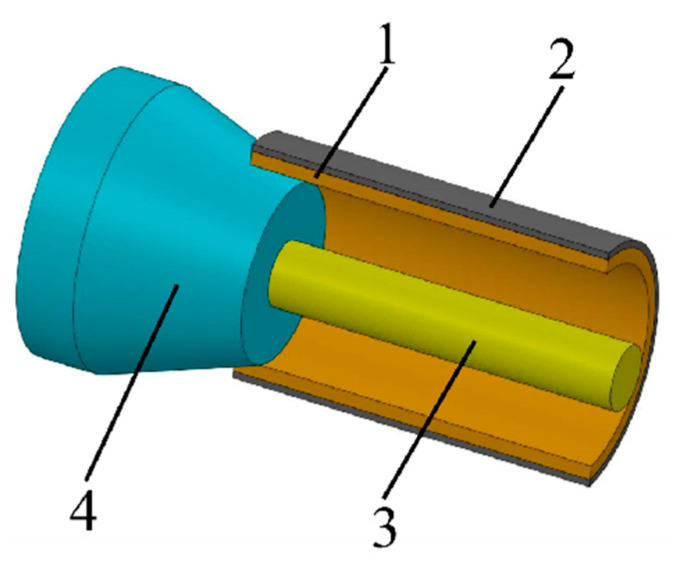
Drawing process (1, inner tube; 2, outer tube; 3, mandrel; 4, drawing die).

**Figure 8:**
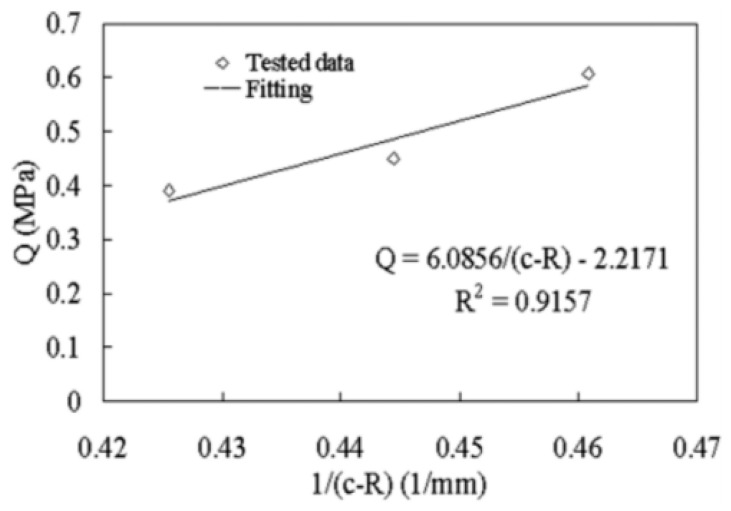
Effect of cone size on interfacial bonding strength [73].

The drawing method combines the inner and outer materials through mold deformation by applying axial force to the composite pipe (Figure 7). In the study, it is found that the cone size directly affects the bonding strength of the composite pipe interface. A larger cone diameter can effectively increase the interface contact area, thereby improving the bond strength and reducing the separation phenomenon caused by stress concentration. By optimizing the cone size in the drawing process, the bonding quality of the inner and outer layers can be significantly improved, and the risk of interlayer separation can be reduced (Figure 8) [73,74,75].

#### 2.4.2. Control of Layer Separation in the Metallurgical Composite Processes

**Figure 9:**
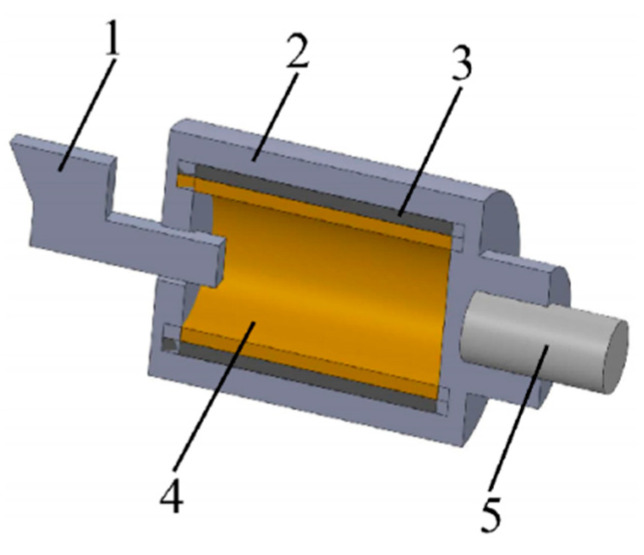
Centrifugal casting process (1, pouring gate; 2, mold; 3, outer tube; 4, inner tube; 5, pindle).

**Figure 10:**
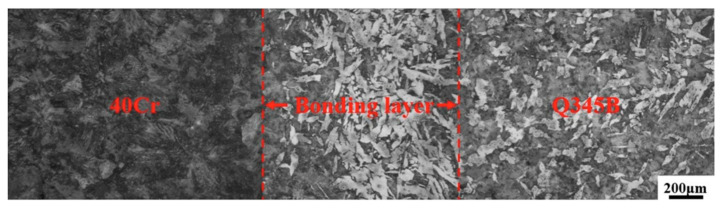
Interfacial microstructure of 40Cr/Q345B bimetallic ring blank [77].

Centrifugal casting utilizes high-speed rotational centrifugal forces to uniformly distribute molten metal along the mold wall, forming a composite structure (Figure 9). Controlling the pouring temperature and mold rotational speed is critical to improving the interfacial bonding quality [76,77,78]. As shown in Figure 10, research on 40Cr/Q345B bimetallic ring blanks in the literature [77] indicates that when the outer metal pouring temperature is 1570 °C and the mold rotational speed is 800 r/min, the axial temperature difference at the interface can be effectively reduced (approximately 75.6 °C), promoting more uniform metallurgical bonding. Furthermore, when the inner metal pouring temperature is 1600 °C and the pouring interval is 221 s, a stable metallurgical bonding layer is formed at the interface, significantly reducing interlayer separation and shrinkage defects. By optimizing these parameters, the diffusion of elements between the inner and outer metals can be effectively enhanced, forming a uniform metallurgical bonding layer at the interface and preventing defects and microcracks caused by excessively fast or slow cooling rates. In addition, reasonable pouring intervals and mold rotational speeds help minimize interface separation caused by thermal stress differences, further enhancing the interfacial metallurgical bonding strength (Table 5).

**Figure 11:**
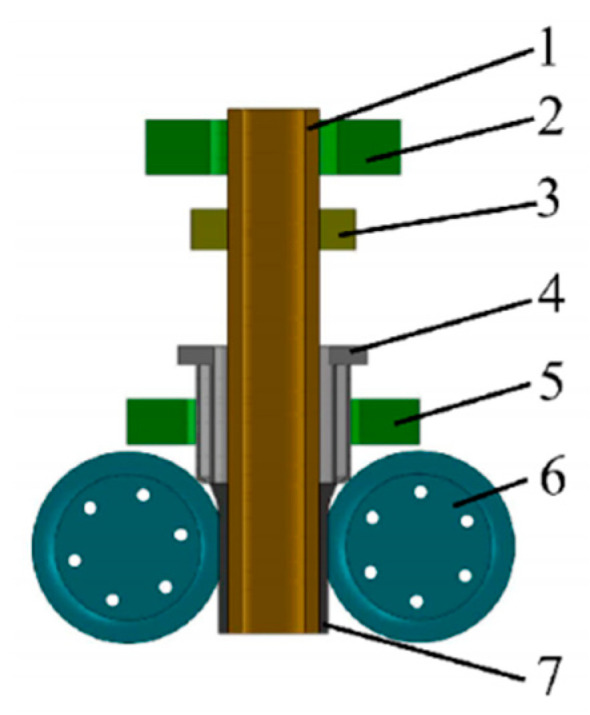
Solid rolling composite process. (1, inner tube; 2 and 5, heating device; 3, guiding device; 4, casting device; 6, cooling rolls; 7, outer tube).

**Figure 12:**
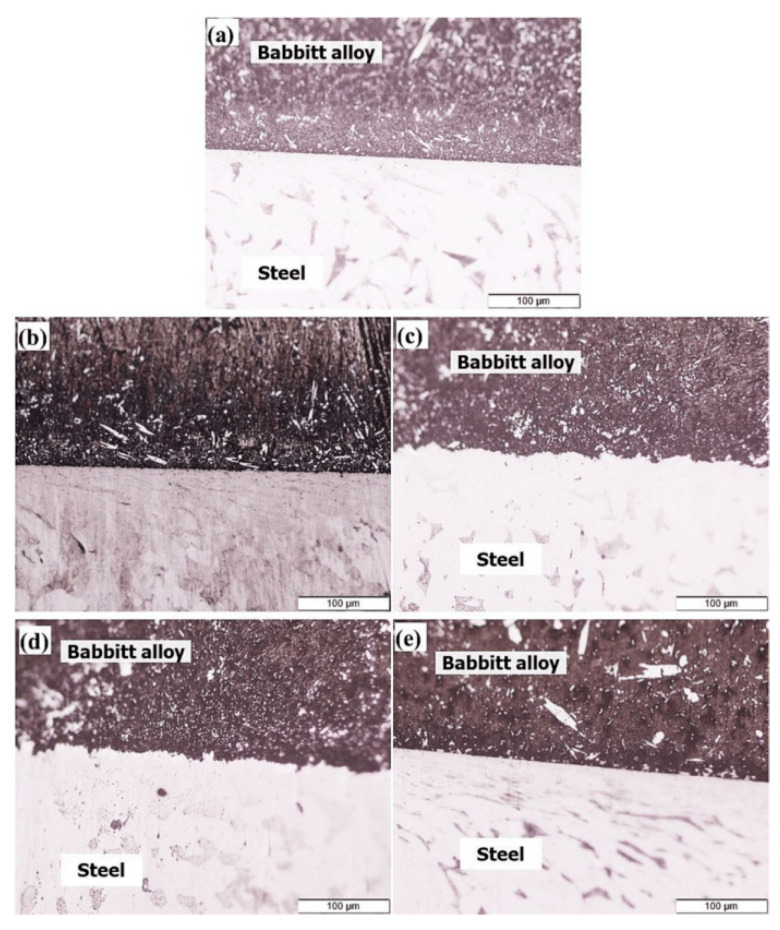
Edge interfacial thickness of Babbitt–steel composite with Sn and Sn–Bi interlayers, (**a**) bimetal with Sn interlayer, (**b**) bimetal with Sn + 1% Bi interlayer, (**c**) bimetal with Sn + 2% Bi interlayer, (**d**) bimetal with Sn + 3% Bi interlayer, and (**e**) bimetal with Sn + 4% Bi interlayer [79].

The solid-liquid casting method forms a bimetallic structure by bonding molten metal with a solid substrate material (Figure 11). Studies [79,80] have shown that introducing an Sn-Bi interlayer can effectively improve interfacial wettability and enhance bonding strength. As observed in Figure 12 [79], the use of an Sn-Bi alloy as an interlayer improves the contact condition between the inner and outer metals, reduces interfacial porosity and inclusions, and prevents interlayer separation caused by interfacial defects.

**Figure 13:**
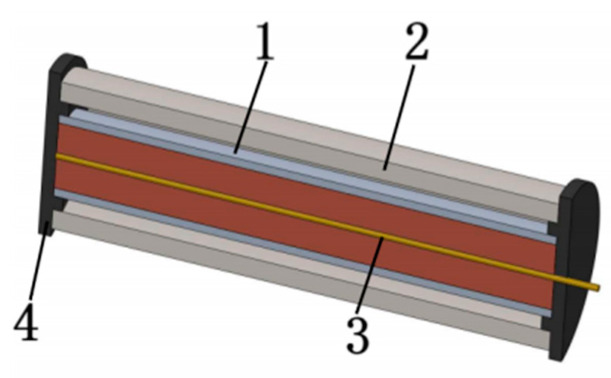
Explosive molding. (1, inner tube; 2, outer tube; 3, detonating cord; 4, piston).

**Figure 14:**
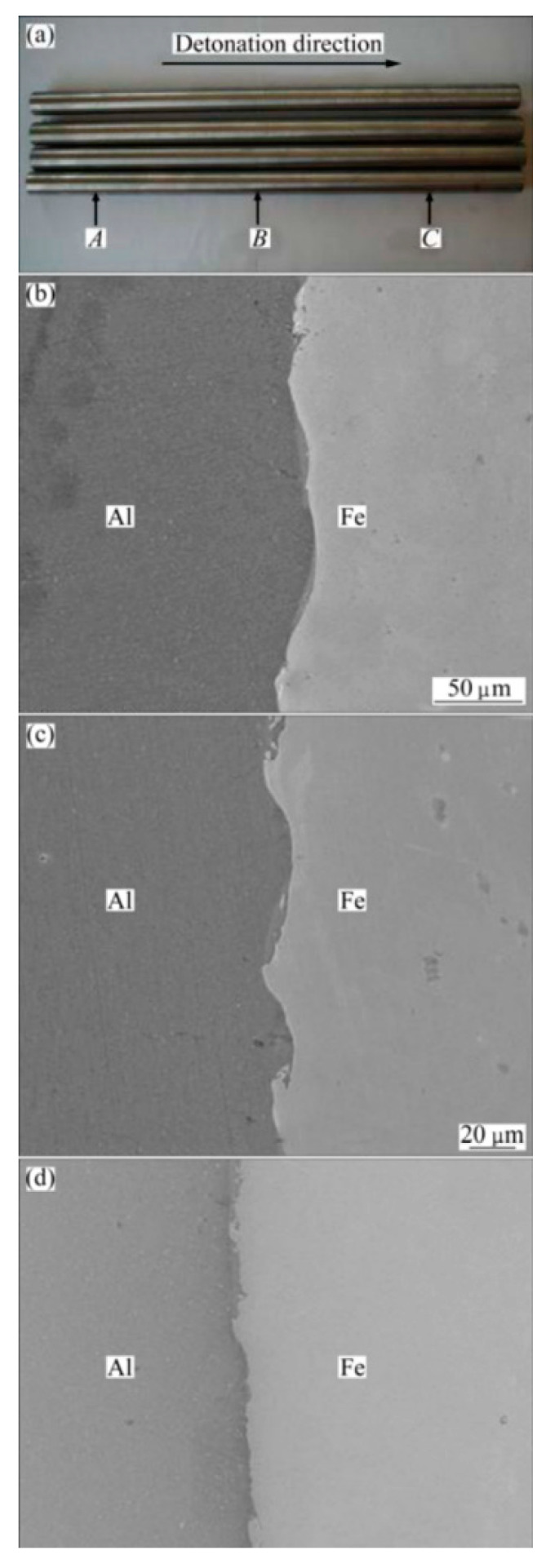
Secondary electron images of interface with good bonding properties: (**a**) sampling positions, (**b**) interface at position A, (**c**) interface at position B, and (**d**) interface at position C [81].

Explosive welding tightly bonds two metals through shock waves and pressure generated by high-energy explosions. Utilizing this technique, effective bonding between the inner and outer materials is achieved under extreme pressure and temperature conditions (Figure 13). As shown in Figure 14, a study [80] on the microstructure of Fe/Al at different detonation points revealed that insufficient detonation energy fails to achieve effective bonding between the materials, while at the terminal position, Fe and Al are effectively bonded. The high pressure and grain refinement induced by explosive welding not only enhance interfacial bonding strength but also effectively suppress the initiation and propagation of interfacial cracks, reducing the occurrence of interlayer separation. Furthermore, optimizing the detonation angle and velocity further improves the metallurgical bonding quality at the welding interface, significantly enhancing the mechanical properties of the composite pipe [30,31,32,81].

### 2.5. Process Optimization Design

Traditional tube bending techniques primarily rely on empirical formulas and limited experimental data, making it difficult to handle complex material and process variations, particularly when addressing nonlinear springback behavior. For example, traditional methods often require extensive trials and adjustments to meet the bending demands of different materials and geometries, which is both time-consuming and costly. Although finite element methods (FEMs) have achieved considerable success in predicting tube bending and forming, their computational complexity limits efficiency, especially in large-scale industrial applications. To overcome the limitations of traditional methods, artificial neural networks (ANNs) and database technologies have been gradually introduced into tube bending processes [82]. ANNs possess the capability to handle complex nonlinear relationships [61], enabling high-precision springback prediction and process optimization, by learning from large datasets of historical data. However, these datasets also present certain limitations: first, the data is primarily derived from simulation results, which, although experimentally validated, may lack sufficient representativeness under extreme conditions; second, the data generation focuses predominantly on specific materials (e.g., aluminum alloys), limiting generalization to other materials; finally, the variation ranges of certain parameters in the datasets are relatively narrow, which may constrain the model’s prediction accuracy, particularly when faced with novel geometries or process conditions (Table 6).

Wu et al. developed a predictive model for the bending deformation of welded thin-walled aluminum alloy square tubes using a backpropagation (BP) neural network [83]. By training the model with 270 sets of finite element simulation data, they successfully demonstrated its ability to predict welding-induced deformation under complex nonlinear conditions with high accuracy. This method not only enhances prediction precision but also minimizes the reliance on physical experiments, thereby significantly reducing production costs. Building on this foundation, Wang et al. highlighted the advantages of artificial neural networks in addressing challenges posed by complex geometries and variable process conditions [84]. They introduced a high-precision rebound prediction model based on a graph neural network (GNN), which achieves an impressive prediction accuracy of 99.993%. The GNN model excels in capturing geometric and process variations in real time during the bending process. Moreover, its real-time feedback capability enables automatic adjustment of process parameters during production, effectively lowering the defect rate and further optimizing manufacturing efficiency.

Database technology played a key role in this process. By constructing and maintaining a database of the pipe bending process, researchers are able to efficiently manage experimental and production data and provide powerful data support for model training. Wu et. al. generated a large amount of training data through finite element simulation and successfully created a dynamically updatable database to optimize the training of the neural network model [83]. This database-driven model training ensures sustainable optimization of the model to maintain highly accurate predictions under changing process conditions.

With the continuous advancement of machine learning and big data technologies, tube bending processes are anticipated to become increasingly intelligent and automated. In particular, the integration of ANN with databases is expected to extend beyond the optimization of production processes to encompass the early stages of design. This development will enable engineers to make real-time predictions and adjustments during product prototyping, thereby enhancing the efficiency and accuracy of product development. Furthermore, tube bending technology is likely to evolve into a more intelligent and interconnected system. Future production processes will seamlessly incorporate real-time monitoring, data analysis, and model optimization, leading to significant improvements in both production efficiency and product quality. By continuously learning from new data, ANN models will be capable of autonomously adjusting production parameters, ensuring that the tube bending process becomes increasingly precise and reliable over time.

**Table 6:** Advantages of the combination of ANN and database.

Advantage	Descriptive
Improved prediction accuracy [85]	Accurate prediction of bending deflection and springback under complex geometric and material conditions
Real-time feedback and process optimization [86]	Provide real-time monitoring and feedback to dynamically adjust process parameters
Reduced costs [87]	Reduces the need for physical experimentation and lowers production and R&D costs

## 3. Bimetallic Composite Pipe Bending Technology

At present, the most widely used bending technologies for bimetallic composite pipes include free bending and CNC bending forming. Compared with monometallic tubes, bimetallic tubes face more complex stress–strain interactions during the forming process, primarily due to the distinct material properties of the inner and outer layers. These differences lead to significant challenges, such as uneven stress distributions, increased springback, and interfacial separation, particularly in high-strength or multi-layered materials. For example, the inner layer may undergo severe compressive deformation, resulting in wrinkling, while the outer layer experiences tensile deformation that increases the risk of cracking. These challenges necessitate strict control of processing parameters [14,66], including neutral layer shift, mold clearance, friction coefficient, and bending angle. In high-strength materials, additional difficulties arise from their higher elastic modulus and yield strength, which exacerbate springback and make precise forming more complex. For multi-layer materials, weak interfacial bonding or differences in thermal and mechanical properties between layers can lead to delamination or interfacial slip during bending.

**Figure 15:**
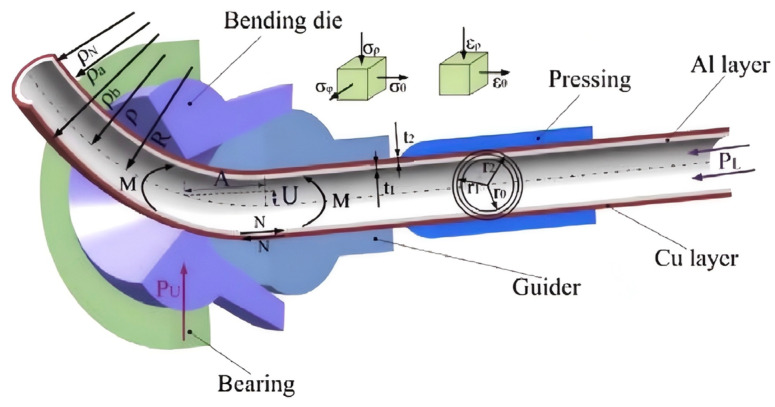
Free bending technology pipeline model [47].

The free bending technique is particularly suitable for composite tube space forming [47] by flexibly adjusting the bending radius and angle [88]. This technique mainly consists of a bending die (Bending Die), a pressing device (Pressing), a guiding device (Guider), and bearings (Bearing). Different bending radii are achieved by replacing or adjusting the radius of the bending die, while the bending angle is controlled by adjusting the position and magnitude of the acting force *P_U_* (as shown in Figure 15). Free bending controls the bending forming process by gradually applying the bending force, which reduces springback and cross-sectional deformation and achieves a more uniform wall thickness distribution without the need for complex molds [89,90,91]. This technique is particularly suitable for the bending forming of bimetallic composite pipes with larger bending radii and smaller diameters, which can significantly improve the forming accuracy and material utilization (Table 7).

**Figure 16:**
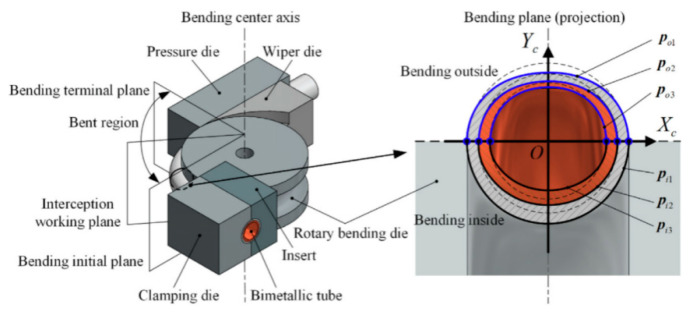
A model combining rotary stretching and bending with numerical control technology [51].

The CNC bending technology for bimetallic composite pipes integrates traditional rotary bending methods with advanced CNC technology, as illustrated in Figure 16. The mold structure used in this process comprises several key components, including the Pressure Die, Wiper Die, Clamping Die, and Rotary Bending Die. The bending force is applied through the Rotary Bending Die to achieve the desired pipe formation. To better understand the forming mechanism and optimize the bending process, extensive research has been conducted using Cu-Ti and Cu-Al bimetallic tubes as experimental models. These studies have demonstrated that the precise formation of bimetallic composite tubes, particularly those with small bending radius and complex three-dimensional geometries, can be achieved by rigorously controlling critical processing parameters. Such parameters include the neutral layer shift, die clearance, friction coefficient, and bending angle [11,44]. As a result, this technology is emerging as a pivotal innovation in the manufacturing of bimetallic composite pipes, offering robust technical support for the large-scale industrial production of intricate pipe fittings [92]. (Table 8).

## 4. Trends in Composite Pipe Bending and Forming

In order to meet the urgent manufacturing needs of high-performance, lightweight bending pipe fittings for industries such as the aerospace and automotive industries, bending theory and technology are showing the following development trends:

(1) Complex geometry and high precision requirements

With the increasing demand for composite fittings, the geometry of pipes also tends to be complex. In recent years, more and more application scenarios require composite fittings to meet the requirements of small bending radii, thin walls, large diameters, and complex three-dimensional shapes. For example, in the automotive and aerospace fields, more complex fluid passages are required. These applications require composite tubes to not only have high mechanical properties but also to enable high-precision bending and forming [93,94]. Therefore, one of the development trends of the composite pipe bending is to improve forming accuracy, especially in the bending process of complex shapes, to maintain uniform wall thickness, stable cross-sectional shapes, and consistent bending radii.

(2) Intelligent process control and automation

Modern manufacturing increasingly relies on intelligent decision-making and automation control, particularly in processes such as composite pipe bending and forming. By integrating advanced technologies, including artificial intelligence (e.g., neural networks), big data analysis, and database systems, it is possible to achieve real-time monitoring and feedback optimization throughout the manufacturing process. For instance, prediction models based on ANN can analyze historical data and dynamically adjust process parameters to maintain high-quality forming, even under complex and variable conditions [95]. This application of intelligent technology not only enhances the efficiency of the bending process but also significantly reduces the scrap rate and lowers production costs [96]. Looking ahead, the deep integration of artificial intelligence with composite pipe bending processes is expected to become the dominant trend in the evolution of composite pipe forming technology.

(3) The development of non-traditional bending technology

In order to cope with the limitations of traditional bending technology, new bending methods are constantly being developed. For example, laser-assisted hot bending, free bending, and CNC bending technology have been gradually applied to the forming process of composite pipe parts [97,98,99]. Laser-assisted hot bending can precisely control the bending angle through localized heating and avoid deformation and defects caused by mechanical contact. Free bending, on the other hand, is able to achieve more flexible tube forming without the use of complex molds, which is particularly suitable for small-batch, high-complexity production needs. With the continuous optimization and improvement of these technologies, they will further improve the flexibility and reliability of the composite pipe forming process.

## 5. Challenges in Composite Pipe Bending and Forming

In response to the above pipe bending trends, the challenges that need to be addressed are summarized below:

(1) Challenges of complex geometry and high precision requirements

With the wide application of bimetallic composite tubes in aerospace, automotive, and other high-precision manufacturing areas, the complex geometry and precision requirements of tube bending have become more stringent. In the bending and forming of small bending radii, thin walls, large diameters, and complex three-dimensional shapes, the differences in stress distribution between the inner and outer materials make the tubes highly susceptible to defects, especially wrinkling of the inner wall, thinning of the outer wall, and cross-sectional distortion [100]. Complex geometric bending increases the difficulty of predicting and controlling these defects, and current theoretical models and process tools are significantly inadequate in dealing with these problems. In addition, as the demand for complex geometries increases, accurately controlling strain-neutral layer offsets during the forming process, as well as coping with inhomogeneous deformations of each layer of material during bending, remain the main challenges for improving forming accuracy.

(2) Challenges of intelligent process control and automation

Although intelligent technologies and automation control are gradually being applied to the bending and forming processes of composite pipes, the realization of complete intelligence still faces many difficulties. Although current artificial intelligence (such as neural networks) and big data analysis have been used to a certain extent for process optimization, these methods require a large amount of data accumulation and algorithm training. They also face the problems of high cost and long cycle times in actual production. Especially when dealing with complex geometries or different material combinations, existing intelligent systems struggle to cope with changing production demands and lack sufficient adaptive capabilities [101]. In addition, there are technical bottlenecks in real-time monitoring and dynamic adjustment of process parameters by automated equipment, which makes it difficult to quickly respond to sudden process changes [102]. Further development of intelligent algorithms and control systems is needed in the future to ensure the efficient and stable operation of process automation.

(3) Development and challenges of non-traditional bending technologies

New bending technologies, such as laser-assisted hot bending, free bending, and CNC bending around the bend, offer new possibilities for the bending and forming of composite pipes. However, practical applications of these technologies still face many problems. Laser-assisted hot bending technology can accurately control the bending angle through non-contact heating, avoiding the deformation defects caused by traditional mechanical contact. However, it has a high sensitivity to material properties and process parameters, especially in bimetallic composite pipe materials with complex interlayer stresses and interfacial bonding. This sensitivity can easily lead to problems such as delamination or cracking [103]. Free bending technology is suitable for small-lot, high-complexity production, but there are still major limitations in high-precision control. Although the CNC bending winding technique is superior in mass production and high efficiency, the precise control of complex multilayer materials and cross-sectional variations still needs to be further optimized. Therefore, how to make breakthroughs in improving the flexibility and adaptability of these new technologies has become a key challenge for the wide application of non-traditional bending technologies.

## 6. Conclusions

This review highlights the critical challenges in bimetallic composite pipe bending, focusing on the limitations of existing methods in addressing defects such as wrinkling, wall thinning, and springback under complex geometries. Compared to previous studies, advancements in computational modeling, including hybrid finite element method-artificial neural network (FEM-ANN) approaches, have significantly improved defect prediction and process optimization accuracy. However, these advancements remain limited by issues such as the quality of input data, the adaptability of algorithms, and challenges in practical implementation.

To overcome these limitations, future research should investigate alternative materials, including lightweight alloys with improved formability, and integrate advanced computational tools like multitask learning (MTL) and digital twin (DT) frameworks for real-time monitoring and process optimization. Additionally, the development of adaptive artificial intelligence (AI)-driven systems capable of dynamic learning under varying manufacturing conditions holds great promise for transforming composite pipe bending techniques. Such innovations could achieve higher precision, efficiency, and product quality, supporting diverse industrial applications.

## Figures and Tables

**Table 1:** Materials and application fields of bimetallic composite pipes in some studies.

Composite Method	Inner Tube Material	Outer Tube Material	Application Field
Mechanical composite	Aluminum Alloys: Al7475, AA5052, Al6063, AA6061, 5A03 Alloy Steel: STKM13C, 316L, SS304	3A21 Aluminum Alloy [26], AZ31 Magnesium Alloy [19], H65 Brass [27], T2 Copper [24] AA1070 Aluminum Alloy [28], 20# Steel [6,25,29]	Chemical pipelines [29], High-temperature cooling walls [30], Solar energy collection [4], Marine development [5], Air conditioning ducts [6], Automotive industry [7]
Metallurgical composite	Aluminum Alloy: Al6061 [18]	1020 Carbon Steel	
	Copper Alloy: T2, C10100	Q235 Carbon Steel [30], Al1070 Aluminum Alloy [31], 6061 Aluminum Alloy [32]	

**Table 2:** Strategies for controlling wrinkling during bending of composite tubes.

Wrinkle Control Strategies	Descriptive
Selection of inner tube material [40,41]	Adoption of inner tube materials with better bending processability and optimization of the ratio of inner and outer tube wall thicknesses
Optimization of process parameters [42,43]	Adjustment of process parameters such as bending radius, bending speed, and temperature to regulate the material stress state
Filled with mandrels [44,45]	Bending process with various filled mandrels (polymers), granular media (sand), or liquid media (water, etc.)
Improvement of initial preparation accuracy [46]	Use of high-precision machining equipment to control the initial preparation process of composite tubes to reduce initial defects and improve molding accuracy

**Table 3:** Strategies for controlling wall thickness reduction and cross-section distortion in the composite pipe bending process.

Wrinkle Control Strategies	Descriptive
Control of material parameters [52,53]	Optimize the ratio of inner and outer tube material and wall thickness to balance the deformation and reduce cross-section distortion
Filled with mandrels [54,55,56]	Filling of bending areas with PVC cores, rigid cores, etc., prevents instability of the cross-section shape
Optimization of process parameters [57,58]	Adjustment of process parameters such as bending angle, lubrication effect, boost speed, bending radius, etc.
Improved bending [59,60]	The use of multi-stage bending, free bending, and other advanced technology to reduce local stress concentration and control wall thickness changes

**Table 4:** Influence factors of springback during bending of composite tubes.

Factor	Law of Influence
Material Properties [63,64,65]	The higher the modulus of elasticity and yield strength, the more pronounced the rebound is
Bending angle [66,67]	The larger the bending angle, the more significant the rebound effect is
Frictional conditions [68,69]	Higher coefficients of friction between the tool and the material result in greater rebound

**Table 5:** Strategies for controlling interlayer separation by different processes.

Crafts	Category	Methods
Spin molding	mechanical compound	Optimization of spinning parameters such as press-in volume, feed rate, etc. to increase residual contact
Drawing		Increased cone diameter for improved interfacial bonding
Centrifugal Casting	Metallurgical composite	Enhanced metallurgical bonding by controlling pouring temperature and mold speed Adjustment of casting intervals and mold speed to improve interfacial element diffusion
Solid-liquid casting		Optimize casting temperature to improve interfacial metallurgical bonding Introduction of Sn-Bi intermediate layer to improve wettability and interfacial bonding
Explosion Welding		Enhanced interfacial bonding through high-pressure conditions created by explosive welding Optimize collision speed and angle, fine crystal enhancement to improve interface integration

**Table 7:** Advantages of free bending.

Evaluation Criteria	Technical Advantages
Accuracy of bending mechanics models	Experimental and theoretical model validation shows that the mechanical model for free bending achieves a prediction deviation of ≤5%, demonstrating high precision and suitability for parameter optimization in complex forming tasks [47,90]
Complex curve forming capability and flexibility	The dynamic adaptability of five-axis and six-axis free bending equipment is high, enabling precise forming of complex three-dimensional paths and multi-radius bends, with errors controlled within ≤0.5 mm [89,91]
Springback prediction and compensation effectiveness	Based on U-R experimental data, springback prediction and compensation techniques for free bending significantly improve forming accuracy. The compensation curve reduces the deviation between the springback angle and the target angle to ≤1° [90]

**Table 8:** Advantages of CNC bending.

Evaluation Criteria	Technical Advantages
Cross-section deformation control capability	The cross-sectional characterization model based on B-spline curve fitting significantly reduces the error compared to traditional elliptic models, with the average cross-sectional deformation error controlled within 1.82% [51]
Bending axis accuracy	The bending axis deviation is controlled within 0.5 mm [51]
Real-time prediction and multitask learning	By integrating multi-source input multitask learning (MTL) with digital twin (DT) technology, real-time prediction of springback and defect classification during the bending process is achieved, enhancing both prediction efficiency and accuracy [92]
